# Part 1: A review of using photovoice as a disability research method: Implications for eliciting the experiences of persons with disabilities on the Community Based Rehabilitation programme in Namibia

**DOI:** 10.4102/ajod.v7i0.418

**Published:** 2018-11-01

**Authors:** Tonderai W. Shumba, Indres Moodley

**Affiliations:** 1 Discipline of Public Health Medicine, University of KwaZulu-Natal, South Africa

## Abstract

**Background:**

Although the Community Based Rehabilitation (CBR) programme in Namibia was formally adopted in 1997, the effectiveness of the programme, including the experiences of persons with disabilities on the programme, has not been assessed to date.

**Objectives:**

To explore the need for a qualitative evaluation tool for the CBR programme that can elicit the experiences of persons with disabilities.

**Methods:**

A scoping review was conducted on the use of photovoice as a disability research method and its potential use in eliciting the experiences of persons with disabilities participating in the CBR programme. A comprehensive literature search was conducted on electronic databases as a part of the scoping review.

**Results:**

Twenty-one studies were selected for review. Six studies followed the exact steps of the traditional photovoice process, and the remaining 15 studies modified the process. Seventeen studies used photovoice as the only research method, 3 combined photovoice with a qualitative method and only one study combined photovoice with a quantitative method. Seven studies had a sample size ranging from 6 to 10 participants as suggested by the traditional photovoice process. The duration of the studies ranged from 2 weeks to 2 years. Thirteen studies investigated life experiences of persons with various disabilities and 17 studies suggested that the photovoice process increases empowerment.

**Conclusion:**

Photovoice is a versatile research method and has the potential to be utilised in effectively eliciting the experiences of persons with disabilities on the CBR programme in Namibia.

## Background

Community Based Rehabilitation (CBR) was initiated by the World Health Organization (WHO) in the early 1980s to improve service delivery for persons with disabilities through providing equal opportunities, social integration, promotion and protection of their human rights (WHO & World Bank 2011). Over the years, CBR has evolved and has been valued as a development approach (Madden et al. 2015). To date, CBR has been implemented in over 90 countries globally (WHO & World Bank 2011).

Community Based Rehabilitation is recommended as one of the best strategies for promoting access to services for persons with disabilities in developing countries (Helander 2007). Further, it has been adopted as a response, in both developed and developing countries, and as a strategy to make available sufficient and appropriate rehabilitation services to a greater number of persons with disabilities (M’kumbuzi & Myezwa 2016). Over the past three decades, CBR has been an effective way of providing care at a local level with integration into primary health care (PHC) for persons with disabilities, particularly those living in remote rural areas.

Despite CBR being in existence for over three decades, its effectiveness from the perspective of persons with disabilities has not been widely assessed. An assessment of the implementation of disability programmes in Namibia from the point of view of both implementers and recipients of services noted that the CBR programme in Namibia is dominated by quantitative data including number of people with disabilities, number of assistive devices distributed, number of persons with disabilities receiving physiotherapy and occupational therapy (Shumba & Moodley 2017). Thus, this study (Shumba & Moodley 2017) recommended the need for a qualitative evaluation tool for the CBR programme that can elicit the experiences of persons with disabilities. These results are consistent with the World Report on Disability that recommended the need for the utilisation of more qualitative methods to investigate the lived experiences of persons with disabilities (WHO & World Bank 2011).

Community Based Rehabilitation evaluation has been conducted at three main levels including community, intermediate and national. At community level, the family plays an important role in providing support and rehabilitation to the person with disability. The intermediate level has professionals linked to government providing support, specialised interventions, training and technical supervision to the community (M’kumbuzi & Myezwa 2016). The government offers the overall planning, coordination and evaluation role to CBR at the national level (Helander 2007). Over the years, at all the levels mentioned above, quantitative methods have allowed for breadth and generalisation of CBR effectiveness. However, quantitative methods are criticised for only providing medically oriented data such as number of persons with disabilities participating in CBR programme, disability types, and assistive devices distributed, but fail to collect data on personal experiences of persons with disabilities. Further, the adoption of positivism in quantitative methods has been criticised for generating findings that are descriptive and lack in-depth analysis of issues. Some researchers proposed the use of various qualitative methods to investigate the experiences of persons with disabilities including focus groups, interviews, document review, questionnaires and nominal groups (Sharma 2004; WHO & IDC 1996). However, these methods have been criticised for relying on the assumptions and judgements of the researcher, and this often results in information which may not reveal the true picture of respondents’ views (Wang & Pies 2004). Further, these conventional methods of data collection may also have the effect of instilling a sense of inferiority and resentment in participants as they often view the researcher as the one processing their thoughts (Wang 1999). It is essential to utilise a tool or process that reflects the unadulterated views and opinions of the individuals with disabilities.

One of the challenges met by the current CBR evaluation frameworks is the low literacy rate amongst persons with disabilities. Low literacy rates amongst persons with disabilities have been revealed in both low-income and high-income countries, with more pronounced patterns in poorer countries (WHO & World Bank 2011). Further, the World Report on Disability revealed that in southern African countries (Malawi, Namibia, Zambia, Zimbabwe), between 24% and 39% of children of 5 years and older with disabilities have never attended school. To this end, there is a need to explore the possibility of using other tools that are more suited to persons with low literacy levels and are participatory in nature.

Participation of persons with disabilities in CBR evaluations can be informative to implementers and policy-makers and can have relevant practical outcomes from the point of view of CBR users. Under the aegis of Article 32 of the UNCRPD, persons with disabilities should be consulted in services in which they are involved (UN 2006). Similarly, Madden et al. (2015) advocated for monitoring systems that are participatory and community owned to ensure programme quality and sustainability. CBR evaluation tools should include persons with disabilities and their community as a central part of the evaluation (Boyce & Ballantyne 2000; Price & Kuipers 2000; WHO & IDC 1996).

The researchers’ preliminary review of literature identified the photovoice method as embracing participatory principles that include persons with disabilities as the central part of the evaluation and as suited for persons with low literacy rates. In this respect, the photovoice method developed by Wang and Burris (1997) appears to have the potential to offer a practical qualitative evaluation tool to elicit the experiences of persons with disabilities on a CBR programme. On the premise that mixed methods allow for triangulation of data in CBR programme evaluation, photovoice can be applied as a qualitative data collection method as well as being incorporated in the monitoring and evaluation frameworks of CBR.

Photovoice is a participatory evaluative tool, commonly used in health research to promote personal and community change for Community-Based Participatory Research (CBPR) because of its accuracy in gathering information (Graziano 2004). Furthermore, photographs captured in photovoice facilitate interpretation of concerns and enable promotion of change (Wang & Burris 1997). However, photovoice has evolved since its initial conceptualisation as a community-based health promotion tool. Photovoice is used as a qualitative research tool for many purposes, including as a participatory evaluation tool (Wang & Burris 1997), a retrospective evaluation method (Kramer et al. 2010) and a needs assessment tool (Findholt, Michael & Davis 2011).

It is therefore important to review the use of photovoice as a disability research method and consider how it can be used for eliciting experiences of persons with disabilities and its role in evaluating the effectiveness of the current CBR programme in Namibia.

## Methods

This scoping review was guided by some aspects of systematic reviews which stipulate that there should be a thorough and transparent process throughout all stages (Mays, Roberts & Popay 2001) and adopted the framework proposed by Arksey and O’Malley (2005). The scoping framework has five stages:

- identification of the research question
- identification of relevant studies
- selecting studies
- charting data
- collating, summarising and reporting results.

We used these stages to guide our scoping review, and the details of each stage are outlined below.

### Identification of the research question

To what extent can published literature provide evidence that photovoice has the potential to effectively elicit the experiences of persons with disabilities? Further, what role can photovoice play in the monitoring and evaluation of a CBR programme?

### Identification of relevant studies

Using the keyword ‘photovoice’ to answer the research question, a search was made in English for all peer-reviewed literature in the following electronic bibliographic databases: PubMed, EBSCOhost (MEDLINE, PsychINFO, Academic Search, Education Source, Health Source), Sage Publication, Science Direct and Web of Science. Reference lists of all included studies were checked to select studies. As some electronic databases may be incomplete, not up-to-date or because abstracting services can vary in coverage, indexing and depth of information (Arksey & O’Malley 2005), hand-searching was also done to identify any unpublished work in local university libraries of Namibia (University of Namibia, National University of Science and Technology, International University of Management), relevant government ministries (Ministry of Health and Social Services, Ministry of Basic Education, Ministry of Poverty Eradication, Ministry of Justice, Office of the Vice President, Office of the Prime Minister) and other relevant agencies (National Disability Council, Office of the Ombudsman).

### Selection of studies

Initially, the key search word was limited to ‘photovoice’ to exclude other types of visual-based methodologies. The researchers were aware that the term ‘photovoice’ was broad and could include a lot of bibliographic references. However, this was important as a starting point to obtain a sense of the volume of literature. Secondly, to narrow down the search and include the relevant articles, the following search words and phrases were used: ‘photovoice AND disability, AND research methods, AND Community Based Rehabilitation’. To eliminate studies that did not address the research questions, the scoping review adopted the inclusion and exclusion criteria indicated below.

#### Inclusion and exclusion criteria

Both researchers reviewed the titles and abstracts and selected articles that provided an indication of where the application of photovoice included persons with disabilities. Where the researchers were in doubt of relevancy, they would review the full articles. Disagreements regarding inclusion were resolved through discussion. The inclusion criteria were original research articles (1) that apply photovoice as a disability research method, (2) that were written in English, (3) that were published between 1997 and 2016, (4) that include all types of study designs (e.g. qualitative, quantitative and mixed methods) to get a broad sense of methodologies that can be combined with photovoice and (5) that used photovoice as a research method with various disability topics that are related to CBR. Preference was given to published articles over dissertations, conference proceedings, organisational reports or manuals when there was duplication of research. The exclusion criteria included (1) articles describing the main use of photovoice for purposes other than research, such as a pedagogical tool or a health promotion intervention (without a research component), (2) original articles that primarily contained descriptive, methodological or conceptual content (vs. empirical) and (3) articles whose photovoice content is duplicated in another source, for example, conference proceedings or theses.

**FIGURE 1:**
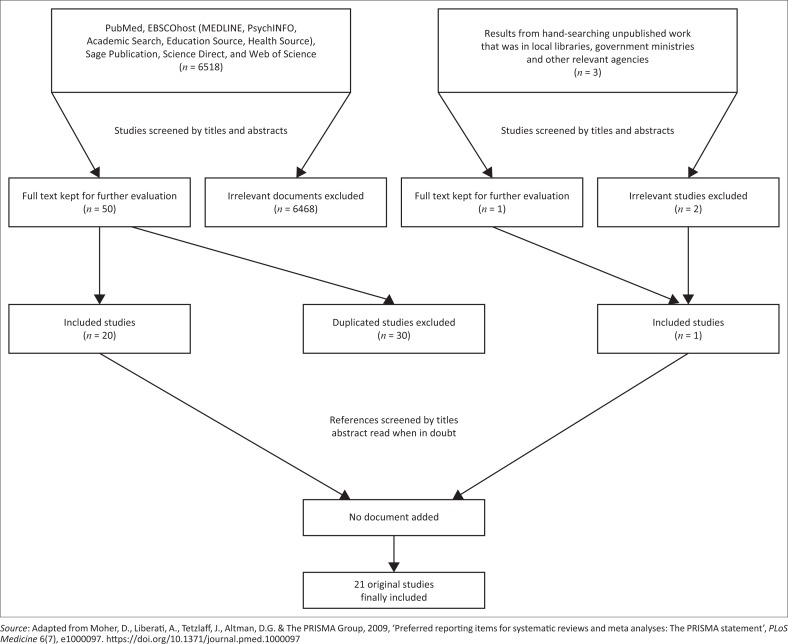
Flow chart for the selection of articles.

The search was conducted in May 2016 and yielded 6518 articles of which 21 met the inclusion criteria. Where a full article was not available, the main author of the article was contacted through email to obtain a reprint of the full article as abstracts may not capture the full scope of an article (Badger et al. 2000). The results of the search and those finally included are shown in Figure 1.

### Charting the data

The final selection of the 21 original research articles was then reviewed and information was compiled on a ‘data charting form’ using an Excel spread sheet. Initially, the following categories of information for each study were recorded: authors, number of studies per year, geographic distribution, research method, sample size, socio-demographic profile, duration of study and types of disability. A limited number of articles had a few of the categories missing. The second author did a blind verification of a random sample of 20% (4) of the articles to check the quality of the categorisation of the charting process. Inconsistencies in the charting process were discussed and resolved.

Secondly, a conventional content analysis (Namey et al. 2008) was undertaken to analyse the purposes, main modifications, methodological challenges and outcomes. This was performed by carefully reviewing the articles, highlighting text that appeared to describe these four areas. These data were extracted verbatim and added to an Excel sheet for coding. The final codes (themes) were examined, followed by a tabulation of frequencies of each theme.

Thirdly, direct content analysis utilising the WHO CBR Matrix (WHO et al. 2010) as a framework to analyse the subject areas addressed by the studies was undertaken. The data were extracted verbatim and added to an Excel sheet for coding using the five components of the WHO CBR Matrix (WHO et al. 2010) as the themes and the corresponding elements as the sub-themes. The five components of the WHO CBR Matrix are health, education, livelihood, social and empowerment. Each component has five elements which represent the focal areas of implementation. The frequencies of these themes and sub-themes were then tabulated.

## Ethical consideration

Ethical approval was obtained from the Human Sciences Research Ethics Committee at the University of KwaZulu-Natal (Ref No. HSS/0646/015D) and approval to collect data was obtained from the Research Committee at the Ministry of Health and Social Services in Namibia (Ref No. 17/3/3).

## Results

### Collating, summarising and reporting the results

A narrative presentation of the results was completed. The numerical analysis of studies included sample size, socio-demographic data, geographical distribution, research methods, types of disability and duration of study. The tables and graphs that were compiled depicted the following: the number of studies published per year, purposes of studies, outcomes and photovoice modifications. This analysis gave a rapid overview and a sense of the main areas of interest and enabled identification of any significant gaps in research.

### Number of studies per year

**FIGURE 2:**
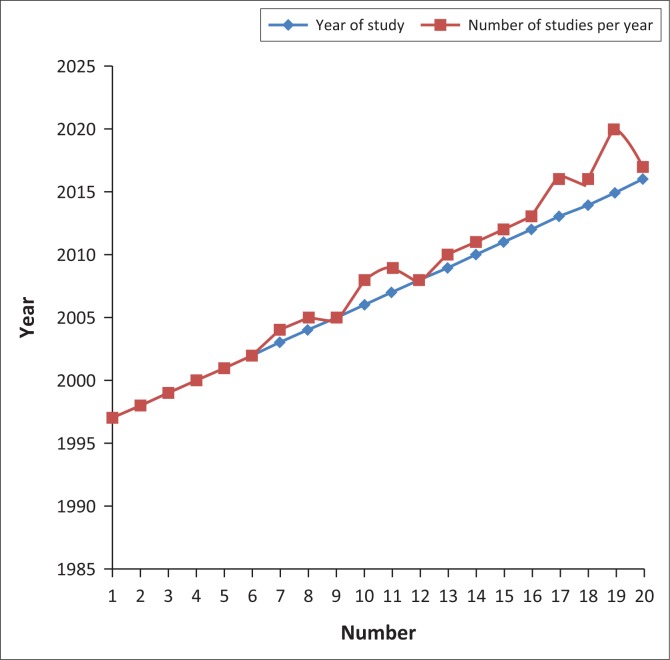
Number of studies per year.

A total of 21 studies were finally selected. From January 1997 to May 2016, the results showed that there was a small but increasing interest in the use of photovoice as a disability research method (Figure 2).

### Geographical distribution

The majority of the studies were conducted in North America (15), with the balance in Africa (3), Europe (2) and Australia (1).

### Research design

Photovoice was used as the sole research design in 17 studies. Four studies (Jurkowski 2008; Ottmann & Crosbie 2013; Russinova et al. 2014; Schleien et al. 2013) combined photovoice with another qualitative method (focus group discussion, diary interview, questionnaire, semi-structured interview, observation, care proxy response). Only one study (Russinova et al. 2014) combined photovoice with mixed-effects regression models.

### Sampling and sample size

In 20 of the 21 studies, participants were purposively selected and only one utilised random selection (Russinova et al. 2014). The sample size ranged from one participant (Bishop, Robillard & Moxley 2013) to 82 participants (Russinova et al. 2014). Seven studies had sample sizes in the range of 6–10 participants (Agarwal et al. 2015; Akkerman et al. 2014; LaDonna & Venance 2015; Newman 2010; Schleien et al. 2013; Shumba, Kloppers & van der Westhuizen 2012; Tijm, Cornielje & Kwaku 2011).

### Socio-demographic profile

Thirteen studies involved mixed genders, one study (Booth & Booth 2003) had only female participants and one study (Clements 2012) had only male participants. Four studies did not define gender. There were 14 studies which used adults over the age of 18 years. Only one study clearly defined using children with ages below 18 years, whereas four studies did not clearly define the age ranges. Two studies had mixed age ranges, that is, adults and children both below and above 18 years old.

### Types of disabilities

Photovoice was used with a range of persons with various disabilities; 10 studies included persons with physical disabilities and eight studies included persons with intellectual disabilities or autism. Photovoice was primarily used directly to elicit the responses of persons with disabilities; however, in one study (Lassetter, Mandleco & Roper 2007), it was used with parents of children with Down’s syndrome and in another (Rampton et al. 2007) with siblings of children with Down’s syndrome. It was also used in one study with persons with visual and speech impairment (Lassetter et al. 2007). Although photovoice relies heavily on visual perception, three studies included persons with visual impairments (Agarwal et al. 2015; Bishop et al. 2013; Cordova et al. 2015).

### Duration of studies

The duration of the reviewed studies ranged from 2 weeks to 2 years. The majority of studies (11) were conducted in periods ranging from 2 weeks to 3 months. Six studies did not define the duration.

### Purpose addressed

The studies had various purposes that they addressed as shown in Table 1. Thirteen of the studies investigated life experiences of persons with different types of disabilities. However, some of studies addressed more than one purpose.

**TABLE 1:** Purposes addressed by studies.

Purpose identified	Number of studies addressing purpose
Promote proactive coping skills	1
Amplify issues of the community	1
Lived experience with regard to community services or programmes	2
Lived experience with regard to community needs assessment or concerns or challenges	2
Lived experience with regard to caregivers	2
Explore the engagement of participants	2
Empower participants	4
Explore perceptions or beliefs or views	4
Gather evidence or identify gaps or document knowledge	5
Lived experience with regard to a disability or illness	7
**Total studies**	**30**

Note: The total articles of the study are 21. However, some of the articles addressed more than one purpose and area.

### Application and modification of Wang and Burris’ (1997) original photovoice methodology

The modifications of the photovoice method that were utilised and corresponding reasons are shown in Table 2. All 21 studies used the methodologies proposed by Wang and Burris (1997) with or without modification. Of the 21 studies, 6 studies followed Wang and Burris’ (1997) traditional photovoice method without any modifications, and the remaining 15 studies modified the method. The main modification implemented was substituting collective group interviewing with individual interviewing of participants.

**TABLE 2:** Application and modification of original photovoice methodology.

Modification	Number of studies	Main reasons for modification
Participants asked to comment on all photos not selecting what they consider as their best photographs summarising their experience	1	When the study is comprehensive, limiting the participants to selecting best photos can hinder the collection and analysis of a wide range of issues (Newman 2010).
Researcher use of International Classification of Functioning (ICF)	1	One of the ICF’s principal uses is to enhance disability advocacy efforts. The ICF facilitates collection and coding of data on environmental factors impeding community participation, whether it is in the presence of a barrier or in the absence of a facilitator (Newman 2010).
Dissemination included a video on disability and two life testimonies from persons with disabilities	1	Real-life stories can be convincing and has the potential of appealing to the emotions of the stakeholders who attend photo galleries (Tijm et al. 2011).
Researcher conducted an anonymous exit survey	1	This was to determine community perception on the photo gallery and community outcomes (Schleien et al. 2013).
Included a step they entitled ‘photographs not taken’ (i.e. they wished to have taken, but failed because of other reasons) at the end of data collection	1	A trusting atmosphere would have been developed between the researcher and the participant and some of the richest data are produced (Lassetter et al. 2007).
Included mixed-effects regression models	1	To examine the impact of photovoice on self-stigma, coping with stigma, empowerment, perceived recovery, self-efficacy and depression (Russinova et al. 2014).
Only one participant was purposively sampled in the study and then snowball sampling was applied to recruit other participants	1	Once a participant assumes ownership, it is easy to recruit others in the photovoice project (Bishop et al. 2013).
Included focus group discussions before recruitment of participants	1	This stimulates participants to start thinking broadly on the issues at hand (Jurkowski 2008).
Used a question guide inspired by Wallerstein and Bernstein’s (1988) question technique	1	Prepares participants to give solutions to the concerns they raise (Jurkowski 2008).
Researcher identified the central purpose of the project and presented it to the participants	1	This was a way to engage participants and a way for them to share their experiences first (Whitney 2006).
Researcher visits sites of each participant’s photographs to confirm	1	Gave insight into the pictures, stories and writing that the students shared with the researcher (Whitney 2006).
Researcher used modified inductive thematic analysis	2	To reflect the individualised experience of the co-researchers (Carpenter & Suto 2008). To orient the research team to a reflective and detailed analysis of each co-researcher’s life experience (Maratos et al. 2016).
Some persons with disabilities had assistants	2	Provides support in the technical aspects of utilising digital cameras, prompts to complete photography assignments and transportation to and from programme meetings or photography locations (Schleien et al. 2013).
Given a minimum number of photos per each given period, for example, at least 10 photos per month	3	To encourage participants to be critical on issues that they want to take photos (Newman 2010).
Developed a follow-up plan after photo gallery	4	To determine impact of photovoice project over a period of time (Bishop et al. 2013).
No collective group interviewing, replaced by individual interviewing with researcher	7	To give participants free expression regarding their pictures in detail and to express their views, not influenced by others, and more depth in their perspective could be gained (Akkerman et al. 2014). To minimise potential transportation issues of participants (Newman 2010). To provide an intimate or safe setting for participants to share their thoughts with the researcher and potentially resulted in much richer interview data (Newman 2010). To gather in-depth views that are not shaped by the perspectives of the other participants (Jurkowski 2008).

### Methodological limitations and challenges

The studies encountered various methodological challenges as shown in Table 3. Almost all studies (18) revealed small sample size as the greatest limitation in allowing for generalisation of results. Other main challenges encountered were as follows: need for assistive technology or assistants for those with more severe disabilities (8 studies), ethics of taking photographs of human subjects (6 studies), communication skills (5 studies), verbal articulation skills in explaining the meaning of photographs (4 studies) and need for sign language interpreters for the deaf (4 studies).

**TABLE 3:** Methodological challenges.

Methodological challenge	Frequency of articles
Persons with disabilities can shun away from photo gallery for fear of public scrutiny	1
Some abstracts aspects like attitude can be difficult to capture or photograph	3
Obsessive tendencies in taking photographs of one item or taking very few photographs resulting in limited picture	4
Some aspects can be absent at the time of photography assignment	4
Photographic censorship applied by parents if employed with children with disabilities or amongst family members	4
Although photovoice is suitable for individuals with low literacy in writing, interpreters are needed in the case of deaf participants	4
Limitation in advocacy skills	5
Ethics of taking photographs of human subjects (procedures for informed consent) proved to be difficult to execute for some participants (cultural sensitivities)	6
Visual images are explanatory but we are still required to provide a written explanation thus challenging on articulation skills	6
Need for assistive technology or assistants for those with more severe disabilities	8
Limited sample size	18

Note: The total number of articles of the study is 21. However, some of the articles had more than one challenge.

### Outcomes of using photovoice with persons with disabilities

**FIGURE 3:**
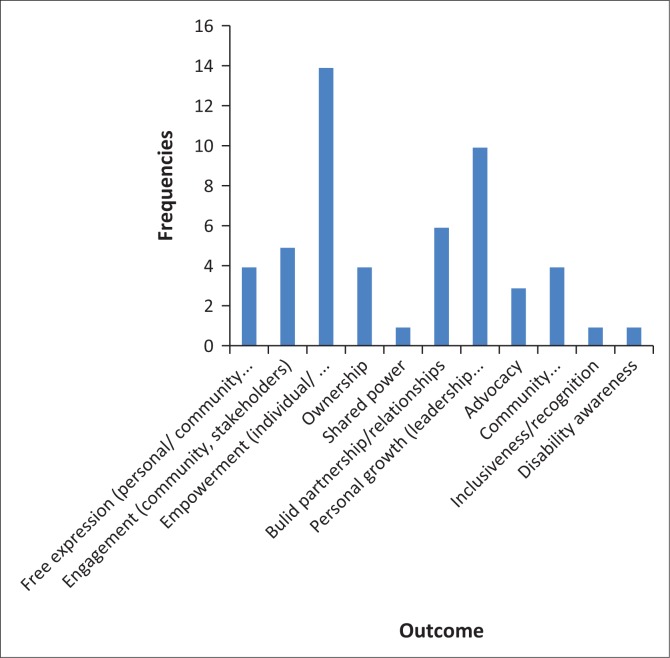
Research outcomes.

Figure 3 shows the range of outcomes achieved by studies and this confirmed consistency with the view that photovoice is an effective tool to be used with persons with disabilities and caregivers for eliciting concerns and communicating these to relevant stakeholders. Seventeen studies revealed that photovoice had a positive effect on empowering persons with disabilities.

### Subject areas addressed

The studies addressed a range of subject areas as shown in Table 4. When the subject areas were analysed using the CBR Matrix (WHO et al. 2010), the studies investigated subject areas that were in line with all the CBR components and corresponding elements. Only two elements were not investigated, including prevention under the health component and culture and arts under the social component. Noteworthy is that all studies addressed the empowerment component with particular focus on communication and social mobilisation. Other study areas that were addressed consistently were personal assistance (11 studies), rehabilitation (8 studies), social protection (7 studies) and self-help groups (5 studies).

**TABLE 4:** Subject areas addressed in line with Community Based Rehabilitation Matrix (WHO et al. 2010).

CBR component	CBR element	Frequencies	Specific areas of study addressed
**Health**	Promotion	3	Physical accessibility to health facilities, access to health and mental health care, health promotion for persons with intellectual disabilities.
	Prevention	0	No articles were found here.
	Medical care	4	Persons with mental illness, physical disabilities.
	Rehabilitation	8	Recovery from mental illness, drug and alcohol abuse, stroke; access to rehabilitation services (physiotherapy, orthopaedic technical services); spinal cord injuries.
	Assistive devices	4	Mobility aids for persons with physical disabilities.
**Education**	Early childhood	1	Early childhood development programmes.
	Primary	3	Inclusion of children with autism, and other developmental disabilities in mainstream schools (inclusive education), communication and physical barriers.
	Secondary and higher	4	Inclusive education for school students with developmental disabilities, university students’ attitudinal and architectural barriers, student empowerment through advocacy skills.
	Non-formal	1	Young persons with physical and sensory impairments.
	Life-long learning	2	Adult learning for persons with disabilities.
**Livelihood**	Skills development	3	Vocational training, on the job training,
	Self-employment	2	Income generating projects.
	Financial services	2	Lack of surety for accessing loans with financial institutions.
	Wage employment	1	Job satisfaction in integrated and sheltered employment.
	Social protection	7	For services such as caregivers, home modifications, rehabilitation professional services, medical aid schemes, disability grant, social housing schemes.
**Social**	Relationship, marriage and family	4	Impact of drug and alcohol abuse on families, experiences of families raising children with developmental disabilities, relationship of siblings of persons with disabilities, caregiver experiences.
	Personal assistance	11	Educational and family assistance of children with developmental disabilities, persons with mental illness, persons with neurological disorders, visual impairments, physical disabilities (wheelchair users).
	Culture and arts	0	No articles were found here.
	Recreation leisure and sports	2	Children with developmental disabilities.
	Access to justice	1	Persons with hearing and visual impairments.
**Empowerment**	Communication	21	Self-advocacy, support for communication skills, address communication barriers, address teaching methods, self-esteem and independence.
	Social mobilisation	21	Community awareness through photo gallery, posters, interaction with community members through photovoice process.
	Political participation	1	Representation of persons with disabilities on community development committees.
	Self-help groups	5	Mothers with intellectual difficulties, community-based mental health rehabilitation agency, community agency serving persons with intellectual disabilities, patients attending a neuromuscular clinic, psychosocial rehabilitation programme.
	Organisations of Persons with Disabilities (OPDs)	1	Development and support to OPDs.

Note: The total articles of the study are 21. However, some of the articles addressed more than one CBR component and element.

## Discussion

The review provided insights on the use of photovoice as a disability research method, the value of utilising it for eliciting the experiences of persons with disabilities and its role in monitoring and evaluating CBR.

A search of original studies on the use of photovoice as a disability research method yielded only 3 studies in Africa, with only one study in Southern Africa. A majority of the articles reviewed were from North America, a similar finding with previous two scoping reviews (Hergenrather et al. 2009; Lal, Jarus & Suto 2012). Further, the review indicated a small but increasing interest in the use of photovoice as a disability research method between 2003 and 2016. The limited use of photovoice as a disability research method in Africa and, in particular, Southern Africa can possibly be attributed to lack of knowledge on the application of the method. We further attribute this to limited research funding allocated to graduate and research programmes in Africa and limited value placed on monitoring and evaluation of disability programmes. Despite limited use of photovoice in the disability field, it is important to note that there is extensive utilisation of photovoice as a research method with many other populations in Southern Africa including HIV and AIDs, maternal health and gender-based violence.

A majority of the studies reviewed (17) utilised photovoice as the only data collection method with only four studies (Jurkowski 2008; Ottmann & Crosbie 2013; Russinova et al. 2014; Schleien et al. 2013) combining photovoice with another qualitative data collection methods (focus group discussion, diary interview, questionnaire, semi-structured interview, observation, care proxy response). Two studies used a mixed method approach with one (Russinova et al. 2014) combining photovoice and quantitative design and the other (Cordova et al. 2015) combining photovoice with CBPR design. The use of photovoice as the only qualitative approach by most studies reviewed in this study seems to be a ‘one-size-fits-all’ approach that is not congruent with the diversity of persons with disabilities and evaluation standards. To this end, evaluation of the CBR programme in Namibia can be enhanced through adding the photovoice method to the already existing quantitative monitoring and evaluation methods. Given that the current quantitative data collection for CBR in Namibia is not comprehensive (Shumba & Moodley 2017), photovoice can potentially enhance the end result of monitoring and evaluation of the CBR programme.

Non-probability (purposive) sampling dominated in most studies reviewed. Notwithstanding the value of purposive sampling, it potentially falls short on selection bias and this may create a threat to the generalisability of the findings. Further purposive sampling does not provide a sample that is representative of the population and thus does not allow transportability of results. To this end, combining purposive sampling and random sampling in a mixed method approach (Russinova et al. 2014) has the potential of enhancing the quality of results in evaluation frameworks.

Sample size ranged from one participant (Bishop et al. 2013) to 82 participants (Russinova et al. 2014). Only seven studies had sample sizes in the range of 6–10 participants (Agarwal et al. 2015; Akkerman et al. 2014; LaDonna & Venance 2015; Newman 2010; Schleien et al. 2013; Shumba et al. 2012; Tijm et al. 2011). The sample size of 6–10 participants is consistent with the sample size originally proposed by Wang and Burris (1997). This flexibility in sample size indicates that photovoice has evolved as a flexible method that can be applied with any sample size depending on the context. The nature of CBR programmes in small communities of Namibia naturally limits the sample size in relation to socio-demographic variables including gender, age groups and disability types, and thus, photovoice is an appropriate research method of choice.

Another insight revealed by this review is that photovoice was used with a range of disabilities. Although photovoice relies heavily on visual perception, three studies included persons with visual impairments (Agarwal et al. 2015; Bishop et al. 2013; Cordova et al. 2015) given that visual impairment covers a spectrum of impairments from low vision to blindness. These studies proved that photovoice is not discriminatory and thus suitable for CBR evaluation that promotes principles of diversity and inclusion.

The duration of the study is usually critical to detect trends in data. This review did not identify a consensus on the desired duration for a photovoice project with the duration of studies ranging from 2 weeks to 2 years. Thus, the duration may be determined by the establishment of set objectives of the study.

With the evolution of photovoice, modifications were suggested to meet the specific aims of different studies. In this review, 15 studies had modifications to the original photovoice process. The main modification in these studies was the replacement of collective group discussion with one-on-one interviews in photovoice analysis, when required. This was to provide participants with confidentiality and freedom of expression when providing narratives regarding their photos without being influenced by others (Newman 2010). Free expression of views by persons with disabilities is one of the principles of the CBR programme (WHO et al. 2010). Furthermore, interviewing persons with disabilities in their homes potentially creates a safe environment that does not only ensure free expression but also enhance confidentiality.

Notwithstanding the potential of utilising photovoice for eliciting the experiences of persons with disabilities, there are some methodological challenges that need to be noted (Table 3). The photovoice process is complex and involves in-depth investigation that validates the use of a small sample. However, small sample sizes have major challenges and limitations of not allowing generalisation of findings. Other critical challenges to address include assistive technology or assistants for those with more severe disabilities, ethics of taking photographs of human subjects, advocacy skills training and the need for sign language interpreters for the deaf.

The conventional content analysis (Namey et al. 2008) of the purposes and outcomes of the studies indicated the value in the use of photovoice method in eliciting the experiences of persons with disabilities. Of the 21 studies reviewed, 13 studies investigated life experiences of persons with various types of disabilities in subject areas of rehabilitation services, community accessibility and caregiver care (Table 1). Further, direct content analysis using the CBR Matrix (WHO et al. 2010) as the framework of analysis indicated that photovoice may be used with persons with disabilities to elicit their experiences regarding education, health, livelihood, social and empowerment (Table 4), which are consistent with the WHO CBR Guidelines (WHO et al. 2010). To this end, photovoice has the potential to be utilised for eliciting the experiences of persons with disabilities with respect to these five components of the CBR Matrix. Thus, these results, though they were not from direct CBR evaluations, indicate the opportunity for using photovoice in monitoring and evaluating CBR in line with the CBR Matrix. The CBR Matrix has the potential of empowering persons with disabilities as they are resolutely underpinned by the UNCRPD (United Nations [UN] 2006) that promotes the social and human rights model of disability.

Under the aegis of Article 31 of the UNCRPD (UN 2006), rehabilitation programmes, including CBR, should be evaluated to determine their effectiveness and relevance. CBR evaluation should not only include quantitative measures such as types of disabilities and number of assistive devises distributed but also include qualitative measures that include the experiences of the beneficiaries of the CBR programme. However, the discourse surrounding evaluating CBR programmes has largely been influenced by the evolution of the concept of disability. The medical model viewed persons with disabilities as patients or recipients of services and their concerns were not solicited or considered to have any merit. With the emergence of social and human rights models, CBR practitioners are required to consult persons with disabilities regarding their opinions and needs. Further, the refrain by persons with disabilities of ‘nothing about us without us’ and Article 32 of the UNCRPD also stipulates that persons with disabilities should be consulted in services in which they are involved (UN 2006). Of the various evaluation tools, photovoice is an appropriate method to elicit qualitative experiences of persons with disabilities to adequately represent the benefits, shortcomings and challenges of CBR programmes. Building on this premise, this study demonstrated that photovoice has the potential to meet the CBR principles of participation, inclusion, sustainability and self-advocacy (WHO et al. 2010) as illustrated in Figure 3. As CBR is the main strategy for delivery of rehabilitation services in Namibia (Government Republic of Namibia 2007), it is essential to have an effective monitoring and evaluation tool and photovoice can meet this requirement.

Photovoice with the suggested modifications to suit specific requirements of disability groups is a practical qualitative evaluation tool that can record the social realities of persons with disabilities that are often not accessible and revealed to CBR evaluators or researchers and are sometimes disregarded by family and community members. Disability data gathered in most Health Information Systems are mostly quantitative and fall short on extracting the true essence of the concerns of persons with disabilities. Thus, findings from photovoice can be used as CBR qualitative indicators that can potentially improve the provision of services and be included in reporting to relevant national and international agencies. Qualitative data complementing quantitative data can lead to the development or evolution of disability policy and legal framework, increasing opportunities of providing policy-makers with evidence of the real concerns of persons with disabilities.

Many persons with disabilities have not benefited from equal educational opportunities and as a result are not as literate as their able-bodied counterparts, thus making use of questionnaires poses a challenge for monitoring and evaluation. Thus, photovoice can help alleviate this challenge especially in areas with a low literacy rate amongst persons with disabilities. Further, photovoice equips persons with disabilities who have limited verbal or expressive abilities with an alternative form of expression, thereby increasing opportunities to engage in the evaluation process (Levin et al. 2007).

CBR programmes aim to empower all persons with disabilities. Historically, persons with disabilities, as many in the apartheid and post-apartheid environment, were not used to having a voice and sharing concerns. Photovoice helps persons with disabilities to identify their concerns, potentially building their confidence through linking them with their peers. Various disability groups benefitting from the CBR programme can possibly have diverse concerns, and photovoice has the potential to help them to come up with a consolidated viewpoint on shared or cross-cutting concerns, as well as identifying those which are specific to their disability.

Wang and Burris (1997) argued that photovoice has a framework that embodies participatory principles. Participatory principles are one of the cornerstones to CBR programming and evaluation. This study mainly focused on the potential role of photovoice as a research method in eliciting the experiences of persons with disabilities. Future studies can investigate the extent to which photovoice can be situated in the context of community participation and/or community-based research including engagement of the participants in the identification of the research purpose or question or problem. Under the aegis of Article 32 of the UNCRPD, persons with disabilities should be consulted in services in which they are involved (UN 2006). This coincides with the vision of the National Policy on Disability (Government Republic of Namibia 1997) that advocates for a ‘Society for All’ that promotes participation and human diversity in one economy.

Despite this study adopting aspects of systematic reviews, there was no attention given to the quality of data. Future research can potentially focus on systematically reviewing the use of photovoice as a disability research tool to strengthen its effectiveness.

## Conclusion

It appears to be clear that CBR practitioners need to explore best practises in CBR for monitoring and evaluation in order to show evidence of the effectiveness of this strategy and to identify areas for improvement. For the CBR programme in Namibia to grow and evolve, the monitoring and evaluation process needs to be adapted to the needs of persons with different disabilities. Photovoice creates an opportunity for persons with disabilities in Namibia to have their concerns heard and documented and ultimately reach policy-makers. Further, photovoice can help alleviate the challenge and inefficiency in utilising the current questionnaire-based monitoring and evaluation of the CBR programme in Namibia, especially in areas with a low literacy rate amongst persons with disabilities. Future research can potentially focus on outcomes of photovoice as a change agent. Additionally, research is needed to establish the conditions under which photovoice can be used to elicit the experiences of persons with disabilities on the CBR programme. The insights proposed in this review may provide guidance on how to use photovoice as a disability research tool and its potential use in CBR monitoring and evaluation utilising the WHO CBR Matrix (WHO et al. 2010).
